# Urban greenspaces harbour distinct plasmid communities enriched in heavy metal resistance and competitive traits in arid soils

**DOI:** 10.1099/mic.0.001705

**Published:** 2026-05-15

**Authors:** María Touceda-Suárez, Alise J Ponsero, Albert Barberán

**Affiliations:** 1 Department of Environmental Science, University of Arizona, Tucson, AZ, USA; 2 Faculty of Medicine, University of Helsinki, Helsinki, Finland; 3 Quadram Institute of Biosciences, Norwich, UK

**Keywords:** arid soils, metagenomics, plasmids, urban greenspaces

## Abstract

Plasmids drive horizontal gene transfer, a fundamental mechanism for soil bacterial evolution and antibiotic resistance emergence. In arid regions, the transformation of natural soils into urban greenspaces introduces dramatic environmental changes that influence the adaptive strategies of soil micro-organisms. Additionally, urban greenspaces can act as interfaces of antibiotic resistance spread between environmental and human microbiomes. Here, we inferred plasmids from soil metagenomes of urban greenspaces in Tucson, AZ, USA, and nearby natural arid habitats. We found urban greenspaces to select for plasmids that carried genes that confer competitive advantages, including motility, prokaryotic defence and resistance to heavy metals. Notably, urban greenspace plasmids exhibited reduced diversity (genetic and functional variants), which could in turn constrain their adaptability to rapid environmental changes. These findings underscore the importance of plasmids as agents mediating soil microbial adaptation to human activities.

## Data Availability

Scripts for the analysis and visualization of data can be found at https://github.com/merytouceda/urban_greenspaces_aridcities. Final data products from processing can be found at 10.5281/zenodo.13152735. Raw metagenomics sequences are available at http://www.ncbi.nlm.nih.gov/bioproject/1143147, reference number PRJNA1143147 (sample accession numbers in Table S1). Supplementary materials can be found at https://doi.org/10.6084/m9.figshare.31079992[1].

## Introduction

Horizontal gene transfer (HGT) – or the exchange of DNA material via mobile genetic elements (MGEs), plasmids and bacteriophages between cells not connected by inheritance – is fundamental for bacterial evolution [2]. For instance, HGT enables rapid microbial adaptation after colonization, exploitation of novel carbon sources and resistance to antimicrobials and heavy metals [3,4]. Importantly, HGT between species from different habitats, primarily driven by plasmids, is the main cause for the emergence of antibiotic-resistant bacteria [5,6].

Although soils, particularly in urban environments, are a significant reservoir of antimicrobial resistance genes, the dynamics of their spread through HGT remain poorly understood [7,8]. Urban greenspaces provide essential ecosystem services, such as air purification, temperature regulation, stormwater management and pathogen control [9], but can also act as interfaces of antibiotic resistance dissemination between environmental and human microbiomes [10]. In arid regions, the transformation of natural soils into urban greenspaces introduces dramatic environmental changes through regular irrigation, fertilization and introduction of non-native plant species to maintain vegetation in an otherwise water-limited environment. These managed parks contrast sharply with surrounding natural arid ecosystems that experience minimal anthropogenic intervention and rely on natural precipitation patterns. Additionally, these alterations could trigger shifts in bacterial life history strategies and in MGE dynamics in relation to antibiotic resistance [11]. In this work, we leveraged 24 soil metagenomes from two urban greenspaces in Tucson, AZ, USA, and 6 nearby natural arid habitats, including shrubland, grassland and ponderosa pine forest, to ask the following: (1) Are there differences in the abundance and diversity of MGEs between urban greenspaces and natural arid soils? (2) Which genes are mobilized in urban greenspaces compared to natural soils, and what patterns of resistance gene abundance emerge from this comparison?

**Fig. 1.**
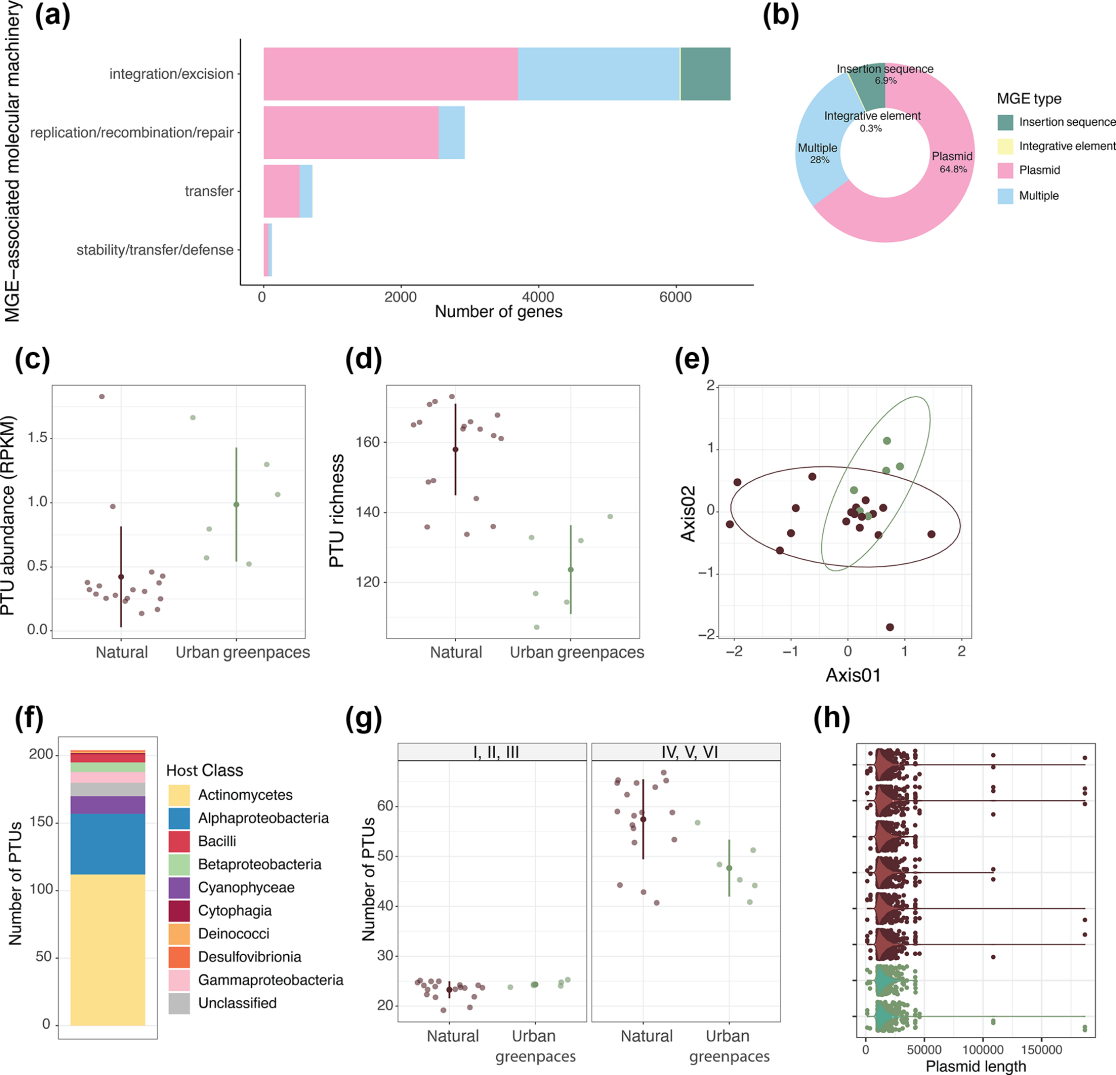
Types of mobile genetic elements and overview of the plasmids in urban greenspaces and natural arid soils. (a) Number of genes associated with the different MGE-associated molecular machinery for each MGE type.** (b)** Number of sequences belonging to the different MGE types. (**c–e)** Abundance, richness and composition of PTUs in urban greenspaces (green) and natural arid soils (brown). Compositional differences are visualized using (NMDS) Non-Metric Multidimensional Scaling ordination of Bray–Curtis dissimilarity matrices (stress=0.09), ‘Axis01’ and ‘Axis02’ labels correspond to NMDS dimensions 1 and 2.** (f)** Number of PTUs associated with host classes. (**g)** Number of PTUs assigned to a host-range grade. PTUs belong to grades I, II and III if they span taxonomic groups within a same family, while they belong to grades IV, V or VI if they span different families.** (h**) Distribution of plasmid length (base pairs) per sample in natural and urban greenspace soils. (c, d, g) Individual data points are shown with jitter. The central point represents the mean, and error bars indicate ±one standard deviation. All samples were collected from the Tucson, Arizona metropolitan area, and surrounding natural habitats within the Sonoran Desert region.

To explore these questions in an arid urban context, we analysed 24 soil metagenomes from urban greenspaces and natural habitats in the Tucson metropolitan area, AZ, USA. This region exemplifies arid urban ecosystems where intensive management is required to maintain vegetation, providing an ideal system to examine how such dramatic environmental modifications influence plasmid ecology. We employed two complementary and independent approaches to identify mobile genetic elements (MGEs). First, we aligned metagenomics assembled contigs against the MobileOG v.2.0 database [12] to survey the diversity of different MGE types across our samples. In parallel, we used geNomad v.1.9.0 [13] to specifically identify plasmid sequences. The MobileOG analysis revealed that plasmids were the most abundant category of MGEs in our dataset (Fig. 1a, b), which, combined with their key role in HGT [14], motivated our decision to focus subsequent analyses on this subset of MGEs. We clustered geNomad-identified plasmid sequences (90% identity and 80% coverage) into 205 plasmid taxonomic units (PTUs) using MMseqs2 v.13.45111 [15]. To limit false detection, only PTUs with a representative sequence longer than 10 kb or with a circular structure (see Methods S1, available in the online Supplementary Material) were retained. We annotated the PTU host taxonomy using Hotspot v.1.1 [16], their host range using HRPredict v.1.0 [17] and the abundance of competitive traits (i.e. cell motility, prokaryotic defence systems and toxin production), xenobiotic resistance genes (XRGs), antibiotic resistance genes (ARGs) and heavy metal resistance genes (HMRGs) using specialized databases and tools (Methods S1).

### Plasmids in urban greenspaces are more abundant but less diverse in natural arid soils

We identified a total of 574 dereplicated sequences carrying MGE genes in our dataset, with the majority carrying plasmid-related genes (64.8%) and MGE-associated genes known to be present in different types of mobile elements (28%, Fig. 1a, b). Given the prevalence of plasmids in the mobilome of our samples, we focused our investigation on the abundance and diversity of plasmids. To capture the plasmid diversity comprehensively, we leveraged an approach that combines homology-based identification (i.e. alignment to reference databases) with sequence-composition-based identification (i.e. using deep learning to find discriminative sequence motifs) [13]. Importantly, only circular sequences were included in this study (see Methods S1 for more information on the plasmid inference process).

Urban greenspace soils showed a significantly higher relative abundance (measured as RPKM or reads per kilobase per million) of PTUs compared to natural soils (*χ*^2^=8.69, *P*-value=0.003; log-transformed: *χ*^2^=13.50, *P*-value<0.001, Fig. 1c). However, urban soils exhibited lower plasmid diversity: observed richness (*χ*^2^=18.68, *P*-value<0.001, Fig. 1d) and Shannon diversity (*χ*^2^=5.15, *P*-value=0.023; log-transformed: *χ*^2^=5.08, *P*-value=0.025), based on rarefied data to account for differences in sequencing depth across samples. Notably, bacterial richness remained unchanged between environments (Fig. S1a). These contrasting patterns – lower diversity and higher relative abundance – indicate that urban greenspace microbial communities harbour fewer distinct plasmid types that make up a larger fraction of the metagenomes (i.e. dominant and abundant plasmids), consistent with differences in plasmid community structure between urban and natural soils. Previous analyses of the microbial communities in these soil samples revealed that bacteria in urban greenspace soils tend to have simplified genomes and to be less versatile due to the higher resource availability [18]. In such conditions, plasmids encoding traits that enhance their host’s competitive fitness or facilitate their own horizontal transfer are expected to persist and proliferate [19]. In contrast, natural arid soils are characterized by a higher heterogeneity, fewer resources and larger fluctuations in temperature and moisture [4,20]. These variable conditions in natural soils could be supporting the presence of a more diverse plasmid pool, as the metabolic cost of maintaining plasmids could be offset by their adaptive benefits during environmental changes, that is, selection for higher genomic versatility [4]. It is important to note that our ‘natural soil’ category encompasses diverse habitat types (arid desert, ponderosa pine forest and shrubland), which may vary in their baseline plasmid diversity. Ponderosa pine forests, with higher moisture and vegetation density, likely represent an upper bound of natural diversity in the region, while desert and shrubland sites represent the drier, sparser end of the spectrum. The observed differences between managed greenspaces and pooled natural sites, therefore, reflect differences in plasmid ecology between intensively managed urban systems vs. the range of natural arid ecosystems found in this region.

The composition of PTUs was significantly distinct between natural and urban greenspace soils (*R*^2^=0.08, *P*=0.014; betadisper: *F*_1,22_=1.71, *P*-value=0.213, Fig. S1c) and moderately correlated with bacterial community composition patterns (Mantel test: *r*=0.31, *P*-value=0.011) (Fig. 1e and Fig. S1b), suggesting that the types and proportions of bacterial species are being influenced by the same environmental factors that are acting on the plasmids. Most predicted plasmid hosts in both environments belonged to the arid soil-dominant bacterial classes *Actinomycetes* and *Alphaproteobacteria* (Fig. 1f), in line with previous reports of plasmid–host associations in the environment [21]. Consistently with the current notions in plasmid mobility across phylogenies [22], most plasmids in our dataset (72%) had a broad host range (grade IV or higher), with predicted hosts spanning across different families and potentially different classes, orders or phyla (Fig. 1g). However, we did not observe any significant differences in the number of plasmids of each host-range grade between natural and urban soils (narrow: *χ*^2^=1.24, *P*-value=0.266; broad: *χ*^2^=2.64, *P*-value=0.104). The prevalence of broad-range plasmids across both soils could suggest their importance for bacterial adaptation in this soil system. While plasmids can be considered selfish or parasitic genetic elements that do not universally confer adaptive traits [23], broad-range plasmids have been reported to often encode antibiotic and heavy metal resistance genes [14,24], which could provide their bacterial hosts with selective advantages in soil.

Despite the significant differences in diversity and composition observed between both ecosystems, plasmids showed similar average lengths in urban and natural soils (Fig. 1h). Plasmid length is associated with their functional capacity, as longer plasmids can carry more accessory traits, but have a higher metabolic cost to their hosts [25,26]. The similar length distributions between natural arid soils and urban greenspaces suggest that plasmids in both ecosystems face comparable trade-offs. Alternatively, our computational approach, which focused on plasmid sequences over 10 kb or with circular structures to minimize the inclusion of false positives, may have also contributed to this similarity in plasmid length by filtering out smaller fragments. This result raises questions about the specific functions encoded by plasmids in both ecosystems and whether the functional repertoire differs between urban and natural arid soils despite their structural similarities.

### Plasmids in urban greenspaces carry more competition and antibiotic resistance genes despite lower functional diversity

**Fig. 2.**
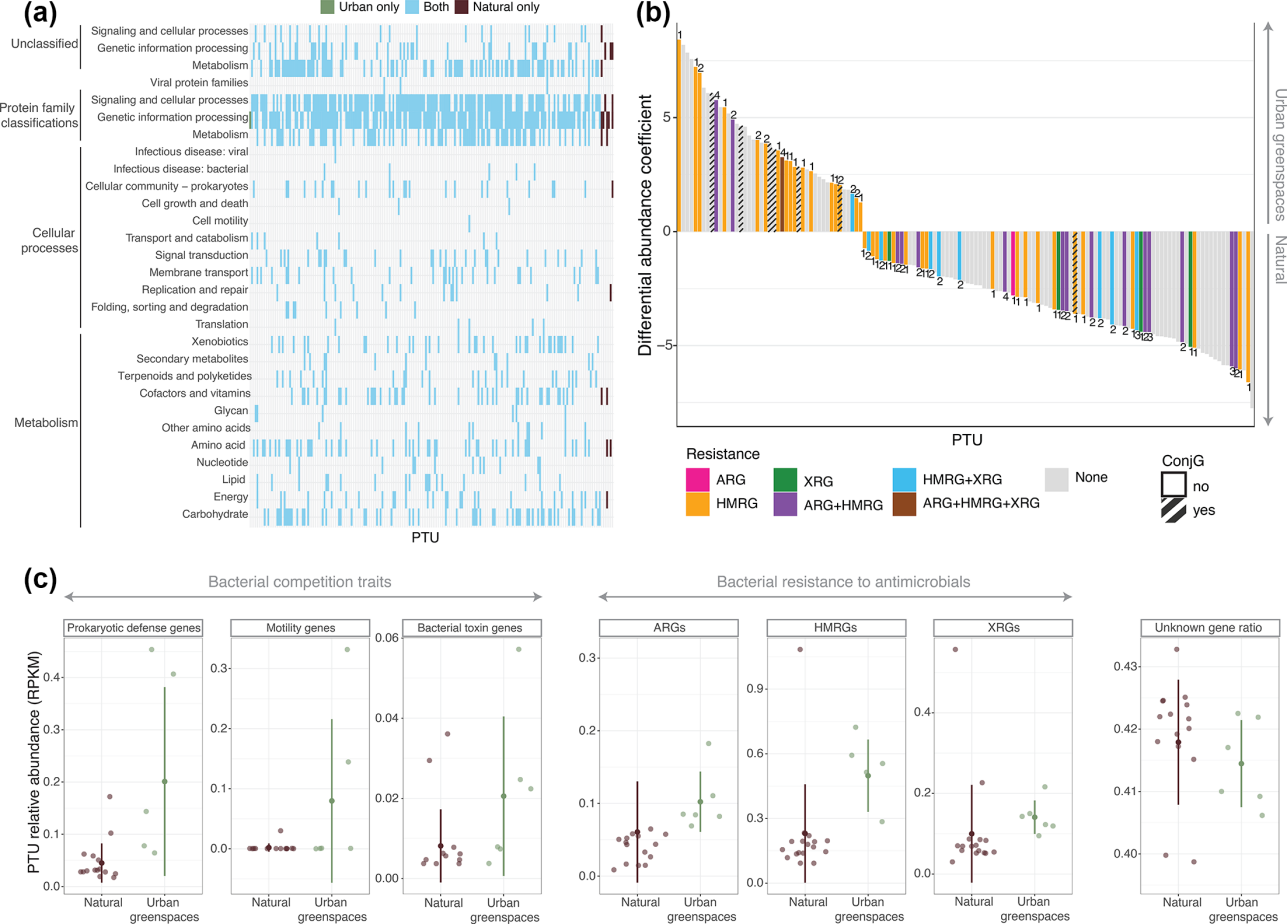
Functional profile of all inferred PTUs, resistance gene presence in differentially abundant PTUs and relative abundance of PTUs with genes/functions of interest urban greenspace and natural arid soils. (a) (KEGG) Kyoto Encyclopedia of Genes and Genomes annotated functional profile of PTUs coloured by their presence in urban greenspaces (green), natural soils (brown) or both (blue). (**b)** Differentially abundant PTUs (upright bar, more abundant in urban greenspaces; downright bar, more abundant in natural soils) and their resistance and conjugation gene content. (**c)** Relative abundance (RPKM) of PTUs associated with different functions in urban greenspaces and natural soils. Central points represent the mean, while error lines represent mean±one standard deviation.

The plasmids present in urban greenspace samples showed a lower functional richness (i.e. number of different orthologous genes, KOs) compared to those detected in natural soils (*χ*^2^=14.16, *P*-value<0.001; log-transformed: *χ*^2^=14.98, *P*-value<0.001, Fig. S2b), while we observed no differences in overall bacterial community functional richness between urban and natural soils (Fig. S2a). This reduction in plasmid functional diversity parallels the lower diversity in PTUs observed in urban soils. As mentioned above, plasmids in both environments had similar average lengths, and urban greenspaces had an average functional to plasmid richness ratio of 5.5 (Fig. 2c), indicating that the observed lower functional diversity is driven by the presence of fewer distinct PTUs rather than a change in the functional capacity of individual plasmids. Furthermore, the functional composition of plasmids was significantly different between urban and natural soils of our dataset (*R*^2^=0.22, *P*-value=0.001; betadisper: *F*_1,22_=0.43, *P*-value=0.531, Fig. S2e, f), mirroring differences observed for the bacterial community functional composition (Fig. S2g, h). This reduction in diversity, together with compositional differences, indicates that urban environments are associated with plasmids carrying specific genetic repertoires.

We found a high relatedness (weighted genome-relative genetic relatedness) between the bacterial and plasmid genetic repertoire (mean of 0.62 out of 1) in both soil environments (*χ*^2^=0.30, *P*-value=0.582, Fig. S2d), suggesting substantial gene exchange between the two populations. Indeed, in both environments, plasmid genes predominantly encoded proteins involved in genetic information processing and signalling and cellular processes (Figs 2a and S3), which are essential for plasmid replication and maintenance [27]. Core bacterial functions such as carbohydrate and amino acid metabolism were also detected in plasmids present in both urban greenspaces and natural soils, supporting previous reports suggesting that core functions are frequently mobilized by plasmids [28]. These metabolic genes, sometimes present in multiple copies [29], provide bacterial hosts with an adaptive advantage to nutrient availability fluctuations [30]. Finally, a large proportion (42%) of all plasmid-encoded genes in both environments lacked any functional annotation (*χ*^2^=0.20, *P*-value=0.655, Fig. 2c). This high percentage of unknown functions, comparable to the 40–60% found in bacterial metagenomes [31], suggests that plasmids are a significant reservoir of novel bacterial functions.

In order to evaluate the potential contribution of plasmids to bacterial adaptation in urban environments, we analysed the relative abundance of PTUs encoding specific competitive and resistance traits. PTUs carrying motility genes (*χ*^2^=4.31, *P*-value=0.038; log-transformed: *χ*^2^=4.06, *P*-value=0.044, Fig. 2c) and prokaryotic defence systems genes (*χ*^2^=5.54, *P*-value=0.019; log-transformed: *χ*^2^=8.59, *P*-value=0.003, Fig. 2c) were significantly more abundant in urban greenspace soils than in natural arid soils. These functions could provide adaptive advantages in the more stable, resource-rich urban greenspace soil conditions where bacterial competition for resources is intensified. Interestingly, we did not observe differences in bacterial (CRISPR) Clustered Regularly Interspaced Short Palindromic Repeats immunity investment between environments nor associations between CRISPR activity and plasmid abundance (Fig. S4), suggesting that changes in this defence mechanism do not explain the higher plasmid prevalence in urban soils [3]. When encoded on a plasmid rather than chromosomes, these traits can be acquired or shed as environmental conditions change, allowing for a rapid adaptation while maintaining compact genomes [18]. In resource-rich urban soils where competition is more intense, but resource inputs are more consistent, the benefits of maintaining these plasmid-encoded competitive functions could outweigh their metabolic costs.

We observed a high prevalence of resistance genes, with 26 out of 205 PTUs (12%) carrying at least one ARG, 101 out of 205 PTUs (47%) carrying HMRGs and 38 out of 205 PTUs (18%) carrying XRGs. ARGs predominantly conferred resistance to tetracycline and fosfomycin (Fig. S5); HMRGs to copper, nickel and iron (Fig. S6); and XRGs to benzoate and polycyclic aromatic hydrocarbons (Fig. S7). We observed a significantly higher relative abundance of plasmids carrying HMRGs (*χ*^2^=6.92, *P*-value=0.009; log-transformed: *χ*^2^=13.82, *P*-value<0.001) and a non-significant trend towards higher abundance for ARGs (*χ*^2^=1.66, *P*-value=0.198; log-transformed: *χ*^2^=2.83, *P*-value=0.092) and XRGs (*χ*^2^=0.65, *P*-value=0.419; log-transformed: *χ*^2^=4.9516, *P*-value=0.026) in urban greenspaces compared to natural soils (Fig. 2c). Notably, our previous metagenomic analyses showed no significant differences in total ARGs and HMRGs relative abundances between environments [18]. This suggests that while the overall prevalence and relative abundance of resistance genes remain similar across environments, likely due to their importance in bacterial survival to diverse environmental conditions [32,33], their mobilization on plasmids is enhanced in urban soils. Further supporting this hypothesis, we found that conjugation genes were significantly enriched in plasmids with higher abundances in urban soils (Fig. 2b), despite resistance genes themselves being ubiquitous across the two environments. This preferential association with mobile elements could contribute to accelerating the spread of antibiotic resistance in urban soil microbial communities and potentially increase transfer to human-associated bacteria [34]. Intriguingly, this result appears contradictory to our findings on host range between urban and arid soils (Fig. 1g), since conjugative plasmids typically exhibit broader host ranges due to their enhanced mobility and transfer capabilities [35]. Future studies employing experimental validation of conjugative transfer and host range determination could help resolve this discrepancy between predicted host range and conjugative potential in our data.

### Study limitations and future directions

Our study focuses on a single metropolitan area in the Sonoran Desert, and we acknowledge that patterns observed here may not directly translate to urban systems in other climatic zones or with different management regimes. The Tucson area represents an arid urban ecosystem where irrigation, fertilization and introduced vegetation create particularly sharp environmental contrasts with surrounding natural habitats. Future comparative studies across multiple cities, climate zones and management intensities will be essential to determine which of our findings reflect general urban soil plasmid dynamics vs. region-specific patterns. Additionally, our natural soil category encompasses habitat diversity (desert, shrubland and forest) that introduces variability within that group. While this heterogeneity limits our ability to attribute differences to specific habitat characteristics, it provides an ecologically realistic baseline representing the range of unmanaged soil conditions in the region. This approach allowed us to test whether urban management effects are detectable even against a diverse natural background, though it precludes strong conclusions about specific natural habitat types. Despite these limitations, our findings provide important baseline data for arid urban soil plasmid ecology – a system type that is both ecologically distinct and geographically expanding as cities grow in water-limited regions worldwide. The patterns we observe – particularly the enrichment of plasmids carrying competitive traits and heavy metal resistance in managed urban soils – warrant investigation across broader geographic scales to assess their generality.

## Conclusion

Altogether, our findings suggest that a distinct group of plasmids may be selected in urban greenspace soils compared to surrounding natural soils. These plasmids carry genes, such as heavy metal resistance, motility and prokaryotic defenses, that could provide the host with an adaptive advantage in the high-resource soil environment artificially created by irrigation and fertilization in water-limited regions. This pattern – higher abundance of specific plasmid types coupled with lower overall diversity – suggests selection for plasmids carrying competitive functions in managed urban environments. While these adaptations might be beneficial in current urban conditions, the lower functional diversity in urban plasmids raises questions about potential constraints to adaptability in the soil microbiome to future environmental changes. Our results from this arid urban system indicate that plasmids could provide a complementary perspective on soil bacterial adaptation to human activities and may contribute to our understanding of microbial responses to environmental changes.

## Supplementary material

10.1099/mic.0.001705 Supplementary Material 1.

## Abbreviations

ARGs: antibiotic resistance genes
HGT: horizontal gene transfer
HMRGs: heavy metal resistance genes
MGEs: mobile genetic elements
PTUs: plasmid taxonomic units
XRGs: xenobiotic resistance genes
