# Supplementary material for: Urban greenspaces harbour distinct plasmid communities enriched in heavy metal resistance and competitive traits in arid soils
Source: Microbiology (Reading). 2026 May 15;172(5):001705. doi: 10.1099/mic.0.001705 (PMC13178938; doi:10.1099/mic.0.001705)
Supplement: Supplementary Material 1. [file mic-172-01705-s001.pdf]

Supplementary Information

Urban greenspaces harbor distinct plasmid communities enriched in heavy metal resistance and competitive traits in arid soils

María Touceda-Suárez[1*], Alise J. Ponsero[2], Albert Barberán[1]

[1] Department of Environmental Science, University of Arizona, Tucson, AZ, USA

[2] Faculty of Medicine, University of Helsinki, Helsinki, Finland

mtoucedasuarez@arizona.edu (Corresponding author)

alise.ponsero@gmail.com

barberan@arizona.edu

Supplementary Methods

Soil sampling and sequencing

As part of a larger study investigating changes in bacterial and viral community structure and genomic potential in arid urban greenspaces, surface soil samples (0–20 cm depth) were collected in Tucson, AZ, USA and surrounding natural areas in August 2019 (Touceda-Suárez et al., 2025). A total of 24 soil samples were collected from eight sites. Six samples were collected in two urban parks—Reid Park (RP; 32°12'28.08'' N, 110°55'24.96'' W, elevation 765 m) and Himmel Park (HP; 32°14'0.42'' N, 110°56'5.48'' W, elevation 752 m)—representing managed urban greenspaces with regular irrigation and maintained vegetation. The remaining 18 samples were collected from six natural areas surrounding the city, representing the regional arid ecosystem diversity: Sonoran desert (Sabino Canyon; SC; 32°18'37'' N, 110°49'16'' W, elevation 830 m), ponderosa

pine forest (Rose Canyon; RC; 32°23'15'' N, 110°42'40'' W, elevation 2,119 m), and arid shrubland at four locations within Santa Rita Experimental Range (range 11B, range 8, Exclosure 45, and UAB; coordinates in Touceda-Suárez et al., 2025). These habitats differ in vegetation and microclimate but share the absence of urban management interventions (irrigation, fertilization, pesticide application, soil amendment, or managed plant communities). We grouped these samples as a 'natural soil' category to test whether urban greenspace management practices create distinct plasmid communities compared to the baseline regional soil diversity. We acknowledge that this grouping encompasses substantial habitat variation (PERMANOVA among natural types: R^2 = 0.3203, p-value = 0.001; betadisper: $F_{2,15}$ = 0.41, p-value = 0.670; Supplementary Figure 8), but this approach allows us to test the conservative hypothesis that urbanization effects are detectable even against a heterogeneous natural baseline. At each site, 9 m transects were established and surface soil samples were collected at three equidistant locations along the transect (3 m, 6 m, and 9 m). Total soil genomic DNA was extracted using a DNeasy PowerLyzer PowerSoil Kit (Qiagen) and shotgun-sequenced on a 2 × 150 bp Illumina NextSeq550 platform at the Microbiome Core of the University of Arizona.

Bacterial taxonomic and functional annotation

Sequenced reads were quality trimmed and cleaned using BBduck v.38.87 (Bushnell, 2022) and Trimmomatic v. 0.38 (Bolger et al., 2014) and assembled into contigs using MEGAHIT v. 1.1.4 (D. Li et al., 2015). Bacterial taxonomy was annotated using Kraken2 v.1.1.1 (Wood & Salzberg, 2014) against the 10/23 RefSeq standard database. A species count table was built using Bracken v.3 (Lu et al., 2017), followed by filtering out of non-

bacterial species and bacterial species with a prevalence of less than 10% of the samples, and an abundance of less than 0.00001. Functional annotation was performed on a sequence catalog of 32,930,898 non-redundant genes inferred with Prodigal and clustered with MMseqs2 v.13.45111(Steinegger & Söding, 2017) at 95% identity and 70% coverage. The gene catalog was annotated against the KEGG database downloaded on December 2024 (Kanehisa et al., 2017) using hmmsearch v.3.3.2 (Johnson et al., 2010). Clean reads were mapped to the gene catalog using BWA v. 0.7.16 (H. Li & Durbin, 2009) and CoverM v.0.6.1 (Aroney et al., 2025) to obtain their abundance per sample. Finally, bacterial average CRISPR investment was calculated as the number of CRISPR spacers in a sample obtained using MinCED v.0.3.2 (https://github.com/ctSkennerton/minced) divided by the number of genomes estimated from the 16S rRNA average copy number (Barnett & Shade, 2024). The 16S rRNA copy number was calculated from clean reads following the methods described in Pereira-Flores et al., 2019.

Mobile genetic element gene annotation

Other mobile genetic elements were identified on contigs through alignment against the Mobile_OG database v.2.0 database (Brown et al., 2022) using BLASTx (McGinnis & Madden, 2004). From the initial 1,175,728 matches between assembled contigs and database only those with percent identity higher than 90% and longer than 50 base pairs were retained. Only the best match between a contig and a mobile_OG identifier was kept. Matches with viral or plasmid sequences were removed to retain contigs exclusively with mobile genetic elements not present in previous analysis, leading to a total of 870

MGE containing contigs, which we clustered at 90% identity and 80% coverage into 574 MGE-containing sequence taxonomic units (MGTUs).

<u>Plasmid inference and annotation</u>

Because of the high number of potential plasmid sequences (Figure 1a-b), and their key role in HGT (Redondo-Salvo et al., 2020) we focused our analysis on plasmid sequences. A total of 64,189 plasmid sequences were inferred using geNomad v.1.9 (Camargo et al., 2023) from 25,665,051 assembled contigs. Inferred plasmid sequences were retained if their length was higher than 10Kb, or if they presented a circular structure, which we assessed by aligning the top and bottom sequences (20% of inferred plasmid sequence length) using BLAST and keeping only alignments with 95% identity and 80% coverage. We obtained 213 plasmid sequences that were grouped into 205 plasmid taxonomic units or PTUs with 90% sequence identity in at least 80% of their sequence. PTU abundance was obtained by mapping clean reads to the PTU sequences using BWA and CoverM. We annotated the PTU taxonomy using Hotspot v.1.1 (Ji et al., 2023), and their host range using HRPredict v.1.0 (Feng et al., 2025), and classified as belonging to grades I,II or III if they spanned taxonomic groups within one host family, or grade IV,V or VI if they spanned across different host families (Redondo-Salvo et al., 2020).

Functional annotation of plasmids followed the same steps and used the same tool and versions as bacterial functional annotation. Briefly, coding sequences were inferred with Prodigal (-f gff -p meta -q -m) v. 2.6 (Hyatt et al., 2010) and clustered with MMseqs2 into a non-redundant gene catalog that was then annotated using hmmsearch (-E 0.00001) v.3.3.2 against the KEGG database (Aramaki et al., 2020). KEGG annotations

were used to study the abundance of traits of interest, such as xenobiotic resistance genes (XRGs; KEGG category: “09111 Xenobiotics biodegradation and metabolism”), and competitive traits: cell motility (KO numbers: "K22221","K02282", "K02282","K02424"), prokaryotic defense systems (includes “defense” in the 3^{d} category level of KEGG), and toxin production (includes “toxin” in the 3^{d} category level of KEGG). Additionally, we annotated antibiotic resistance genes (ARGs) using Abricate v.1.0.1 (Seemann, n.d.) against NCBI (Feldgarden et al., 2019), geNomad-embedded NCBIfam-AMRFinder v.4.0 (Feldgarden et al., 2021); and heavy metal resistance genes (HMRGs) using DIAMOND v.2.0.9 (Buchfink et al., 2015) against the BacMet v.2.0 database (Pal et al., 2014). Finally, we included manually selected Prokka annotations that referred to antibiotic and heavy metal resistance genes (Prokka descriptions containing keywords like “resistance”, “ars”, “Beta-lactamase”; for full list see mges/scripts/analyses/ptus_analyses.R) in the differential abundance calculations for resistance genes (see below). Conjugation genes were annotated in the plasmid inference process by geNomad.

Statistical analyses

Statistical analyses were implemented in R v4.2.2 (R Core Development Team, 2015). Alpha diversity measures (i.e. richness, Shannon diversity, and evenness) were calculated using the *vegan* package v.2.6-4 (Oksanen et al., 2020) on rarefied data to account for differences in sequencing depth across samples. The differences in alpha diversity measures and abundance of plasmids between urban greenspaces and natural soils were evaluated using a mixed-effects model with site as a random effect using *lme4*

(Bates et al., 2015). Log transformation was applied to measures with non-normal residuals. Read counts were normalized to RPKM (reads per kilobase per million) using the formula: RPKM = (count × 10^9) / (plasmid length × total reads), and the resulting values were used to calculate a Bray-Curtis dissimilarity matrix. We implemented PERMANOVA to test the effect of urbanization on plasmid composition while controlling for site effects and used Mantel tests to compare differences in bacterial and plasmid composition using the vegan package. Homogeneity of dispersions was confirmed using the *vegan* function *betadisper*. Additionally, we calculated the weighted gene repertoire relatedness (wGRR), - defined as the mean of gene families present in both plasmids and bacteria – as the sum of the identity between MMseqs2-aligned best bi-directional (or reciprocal) hits divided by the number of gene families in the group with fewer families (Rocha, 2018). Differential abundance of plasmids carrying resistance genes was calculated using the Maaslin2 package v.1.12.0 using the same mixed-effects model (Mallick et al., 2021).

References

Aramaki, T., Blanc-Mathieu, R., Endo, H., Ohkubo, K., Kanehisa, M., Goto, S., & Ogata, H. (2020). KofamKOALA: KEGG Ortholog assignment based on profile HMM and adaptive score threshold. *Bioinformatics (Oxford, England)*, *36*(7), 2251–2252. https://doi.org/10.1093/bioinformatics/btz859

Aroney, S. T. N., Newell, R. J. P., Nissen, J. N., Camargo, A. P., Tyson, G. W., & Woodcroft, B. J. (2025). CoverM: Read alignment statistics for metagenomics.

Bioinformatics (Oxford, England), *41*(4), btaf147. https://doi.org/10.1093/bioinformatics/btaf147

Barnett, S. E., & Shade, A. (2024). *Soil viral community dynamics over seven years of heat disturbance: Spatial variation exceeds temporal in annually sampled soils* (p. 2024.05.27.596044). bioRxiv. https://doi.org/10.1101/2024.05.27.596044

Bates, D., Mächler, M., Bolker, B., & Walker, S. (2015). Fitting linear mixed-effects models using lme4. *Journal of Statistical Software*, *67*, 1–48. https://doi.org/10.18637/jss.v067.i01

Bolger, A. M., Lohse, M., & Usadel, B. (2014). Trimmomatic: A flexible trimmer for Illumina sequence data. *Bioinformatics*, *30*(15), 2114–2120. https://doi.org/10.1093/bioinformatics/btu170

Brown, C. L., Mullet, J., Hindi, F., Stoll, J. E., Gupta, S., Choi, M., Keenum, I., Vikesland, P., Pruden, A., & Zhang, L. (2022). mobileOG-db: A manually curated database of protein families mediating the life cycle of bacterial mobile genetic elements. *Applied and Environmental Microbiology*, *88*(18), e00991-22. https://doi.org/10.1128/aem.00991-22

Buchfink, B., Xie, C., & Huson, D. H. (2015). Fast and sensitive protein alignment using DIAMOND. *Nature Methods*, *12*(1), Article 1. https://doi.org/10.1038/nmeth.3176

Bushnell, B. (2022). *BBMap*. SourceForge. https://sourceforge.net/projects/bbmap/

Camargo, A. P., Roux, S., Schulz, F., Babinski, M., Xu, Y., Hu, B., Chain, P. S. G., Nayfach, S., & Kyrpides, N. C. (2023). Identification of mobile genetic elements with geNomad. *Nature Biotechnology*, 1–10. https://doi.org/10.1038/s41587-023-01953-y

Feldgarden, M., Brover, V., Gonzalez-Escalona, N., Frye, J. G., Haendiges, J., Haft, D. H., Hoffmann, M., Pettengill, J. B., Prasad, A. B., Tillman, G. E., Tyson, G. H., & Klimke, W. (2021). AMRFinderPlus and the reference gene catalog facilitate examination of the genomic links among antimicrobial resistance, stress response, and virulence. *Scientific Reports*, *11*(1), 12728. https://doi.org/10.1038/s41598-021-91456-0

Feldgarden, M., Brover, V., Haft, D. H., Prasad, A. B., Slotta, D. J., Tolstoy, I., Tyson, G. H., Zhao, S., Hsu, C.-H., McDermott, P. F., Tadesse, D. A., Morales, C., Simmons, M., Tillman, G., Wasilenko, J., Folster, J. P., & Klimke, W. (2019). Validating the AMRFinder tool and resistance gene database by using antimicrobial resistance genotype-phenotype correlations in a collection of isolates. *Antimicrobial Agents and Chemotherapy*, *63*(11), e00483-19. https://doi.org/10.1128/AAC.00483-19

Feng, T., Chen, X., Wu, S., Tang, W., Zhou, H., & Fang, Z. (2025). Predicting the bacterial host range of plasmid genomes using the language model-based one-class support vector machine algorithm. *Microbial Genomics*, *11*(2), 001355. https://doi.org/10.1099/mgen.0.001355

Hyatt, D., Chen, G.-L., LoCascio, P. F., Land, M. L., Larimer, F. W., & Hauser, L. J. (2010). Prodigal: Prokaryotic gene recognition and translation initiation site identification | BMC Bioinformatics | Full Text. *BMC Bioinformatics*, *11*(119). https://bmcbioinformatics.biomedcentral.com/articles/10.1186/1471-2105-11-119

Ji, Y., Shang, J., Tang, X., & Sun, Y. (2023). HOTSPOT: Hierarchical host prediction for assembled plasmid contigs with transformer. *Bioinformatics*, *39*(5), btad283. https://doi.org/10.1093/bioinformatics/btad283

Johnson, L. S., Eddy, S. R., & Portugaly, E. (2010). Hidden Markov model speed heuristic and iterative HMM search procedure. *BMC Bioinformatics*, *11*(1), 431. https://doi.org/10.1186/1471-2105-11-431

Kanehisa, M., Furumichi, M., Tanabe, M., Sato, Y., & Morishima, K. (2017). KEGG: New perspectives on genomes, pathways, diseases and drugs. *Nucleic Acids Research*, *45*(D1), D353–D361. https://doi.org/10.1093/nar/gkw1092

Li, D., Liu, C. M., Luo, R., Sadakane, K., & Lam, T. W. (2015). MEGAHIT: An ultra-fast single-node solution for large and complex metagenomics assembly via succinct de Bruijn graph. *Bioinformatics*, *31*(10), 1674–1676. https://doi.org/10.1093/bioinformatics/btv033

Li, H., & Durbin, R. (2009). Fast and accurate short read alignment with Burrows-Wheeler transform. *Bioinformatics (Oxford, England)*, *25*(14), 1754–1760. https://doi.org/10.1093/bioinformatics/btp324

Lu, J., Breitwieser, F. P., Thielen, P., & Salzberg, S. L. (2017). Bracken: Estimating species abundance in metagenomics data. *PeerJ Computer Science*, *3*, e104. https://doi.org/10.7717/peerj-cs.104

Mallick, H., Rahnavard, A., McIver, L. J., Ma, S., Zhang, Y., Nguyen, L. H., Tickle, T. L., Weingart, G., Ren, B., Schwager, E. H., Chatterjee, S., Thompson, K. N., Wilkinson, J. E., Subramanian, A., Lu, Y., Waldron, L., Paulson, J. N., Franzosa, E. A., Bravo, H. C., & Huttenhower, C. (2021). Multivariable association discovery

in population-scale meta-omics studies. *PLOS Computational Biology*, *17*(11), e1009442. https://doi.org/10.1371/journal.pcbi.1009442

McGinnis, S., & Madden, T. L. (2004). BLAST: At the core of a powerful and diverse set of sequence analysis tools. *Nucleic Acids Research*, *32*(Web Server issue), W20–W25. https://doi.org/10.1093/nar/gkh435

Oksanen, J., Blanchet, G. F., Friendly, M., Kindt, R., Legendre, P., McGlinn, D., Minchin, P. R., O'Hara, R., Simpson, G., Solymos, P., Stevens, M., Szoecs, E., & Wagner, H. (2020). *Vegan: Community Ecology Package. R package version 2.5-6.* [Computer software].

Pal, C., Bengtsson-Palme, J., Rensing, C., Kristiansson, E., & Larsson, D. G. J. (2014). BacMet: Antibacterial biocide and metal resistance genes database. *Nucleic Acids Research*, *42*(Database issue), D737–D743. https://doi.org/10.1093/nar/gkt1252

Pereira-Flores, E., Glöckner, F. O., & Fernandez-Guerra, A. (2019). Fast and accurate average genome size and 16S rRNA gene average copy number computation in metagenomic data. *BMC Bioinformatics*, *20*(1), 453. https://doi.org/10.1186/s12859-019-3031-y

R Core Development Team. (2015). R: a language and environment for statistical computing, 3.2.1. *Document Freely Available on the Internet at: Http://Www. r-Project. Org*. http://www.r-project.org/

Redondo-Salvo, S., Fernández-López, R., Ruiz, R., Vielva, L., de Toro, M., Rocha, E. P. C., Garcillán-Barcia, M. P., & de la Cruz, F. (2020). Pathways for horizontal gene

transfer in bacteria revealed by a global map of their plasmids. *Nature Communications*, *11*(1), 3602. https://doi.org/10.1038/s41467-020-17278-2

Rocha, E. P. C. (2018). Neutral Theory, Microbial Practice: Challenges in Bacterial Population Genetics. *Molecular Biology and Evolution*, *35*(6), 1338–1347. https://doi.org/10.1093/molbev/msy078

Seemann, T. (n.d.). *Abricate* [Computer software]. https://github.com/tseemann/abricate

Skennerton, C. T. (2025). *MinCED - Mining CRISPRs in environmental datasets* [Java]. https://github.com/ctSkennerton/minced (Original work published 2013)

Steinegger, M., & Söding, J. (2017). MMseqs2 enables sensitive protein sequence searching for the analysis of massive data sets. *Nature Biotechnology*, *35*(11), Article 11. https://doi.org/10.1038/nbt.3988

Touceda-Suárez, M., Ponsero, A. J., & Barberán, A. (2025). Differences in the genomic potential of soil bacterial and viral communities between urban greenspaces and natural arid soils. *Applied and Environmental Microbiology*, *91*(8), e0212424. https://doi.org/10.1128/aem.02124-24

Wood, D. E., & Salzberg, S. L. (2014). Kraken: Ultrafast metagenomic sequence classification using exact alignments | Genome Biology | Full Text. *Genome Biology*, *15*. https://genomebiology.biomedcentral.com/articles/10.1186/gb-2014-15-3-r46

Supplementary Figures and Tables

Supplementary Figure 1. Bacterial community structure in urban greenspaces and natural soils. a. Bacterial species richness, or number of different bacterial species χ^2 =

0.42, p-value = 0.515. Individual data points are shown with jitter. The central point represents the mean, and error bars indicate ± one standard deviation. **b.** Bacterial community composition (R^2 = 0.20, p-value = 0.005) visualized using NMDS ordination of Bray-Curtis dissimilarity matrices. **c**. PCoA ordination showing group centroids and individual sample dispersion (left), and boxplots of distances to centroids for each group (right).

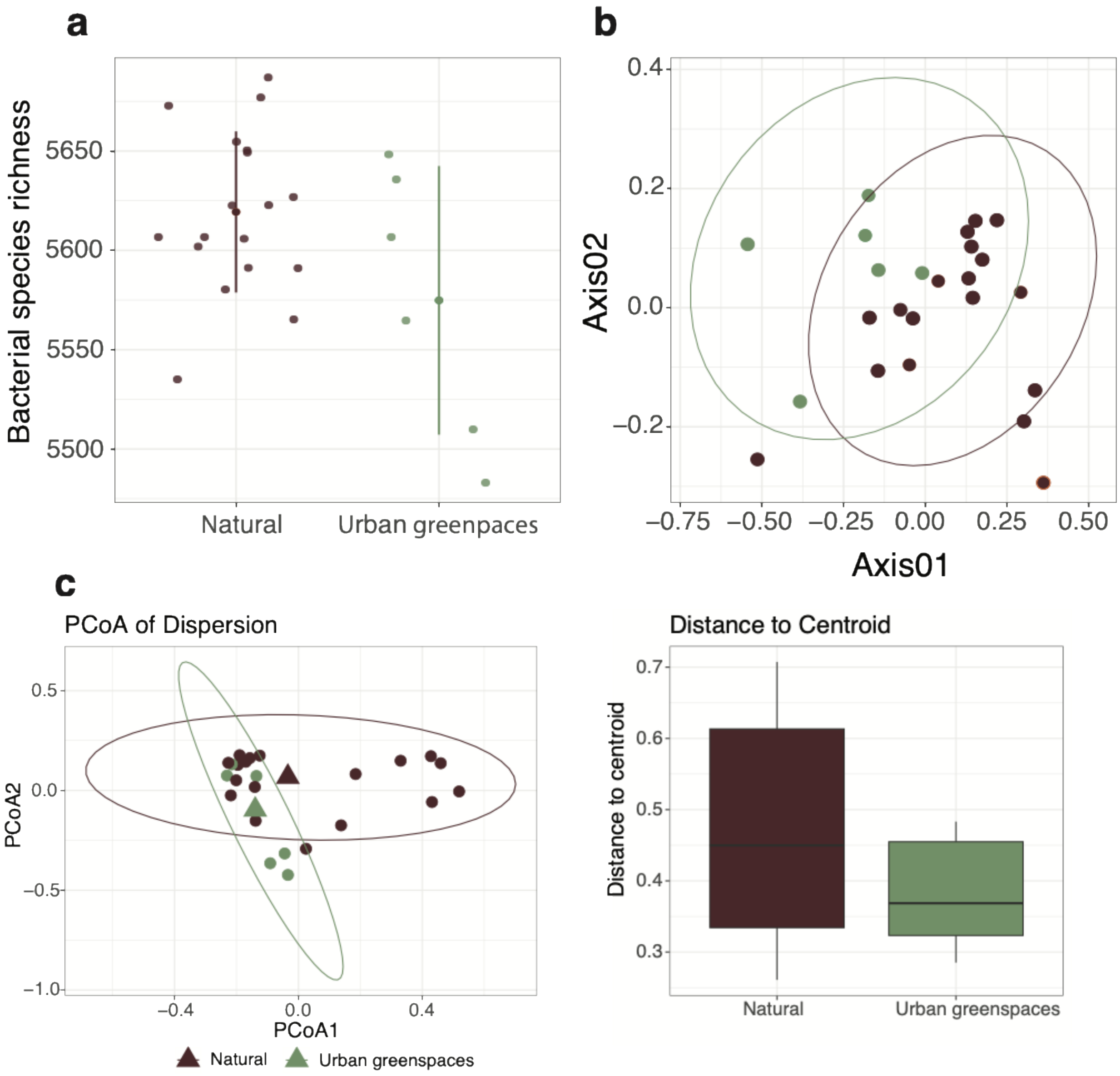

Supplementary Figure 2. Differences in the functional richness, and composition between bacteria and plasmids in urban greenspaces and natural arid soils. a-b. Functional richness, or number of different genes in bacteria (χ^2 = 0.42, p-value = 0.515) and plasmids (χ^2 = 14.03, p-value < 0.001). **c.** Ratio between bacterial and plasmid functional richness (χ^2 = 6.11, p-value = 0.013). **d.** Weighted gene repertoire relatedness between bacteria and plasmids (χ^2 = 0.30, p-value = 0.582). Individual data points are shown with jitter, central points represent the mean while error lines represent mean ± one standard deviation. **e-f**. PCoA ordination of Bray-Curtis dissimilarity showing group centroids and individual sample dispersion (e), and boxplots of distances to centroids for each group (f). **g-h.** Bacterial **(**R^2 = 0.52, p-value = 0.001) and plasmid (R^2 = 0.22, p-value = 0.001) functional composition visualized using NMDS ordination of Bray-Curtis dissimilarity matrices. **Panels a-d:** Individual data points are shown with jitter. The central point represents the mean, and error bars indicate ± one standard deviation.

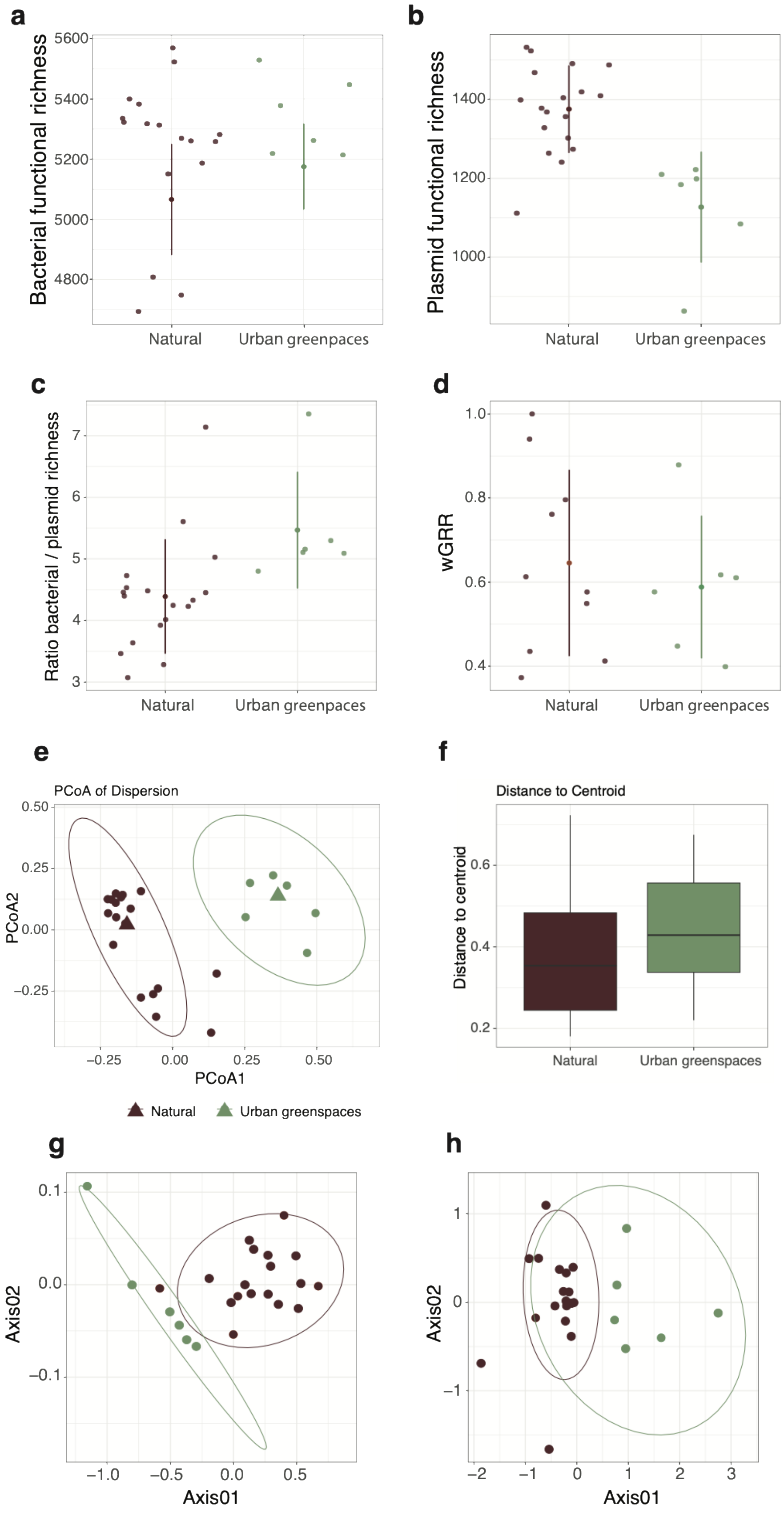
a
Bacterial functional richness
Natural
Urban greenpaces
b
Plasmid functional richness
Natural
Urban greenpaces
c
Ratio bacterial / plasmid richness
Natural
Urban greenpaces
d
wGRR
Natural
Urban greenpaces
e
PCoA of Dispersion
PCoA2
PCoA1
Natural
Urban greenspaces
f
Distance to Centroid
Distance to centroid
Natural
Urban greenspaces
g
Axis02
Axis01
h
Axis02
Axis01

Supplementary Figure 3. KEGG annotated functional profile of PTUs, ordered through hierarchical clustering and colored by their presence in urban greenspaces (green), natural soils (brown), or both (blue).

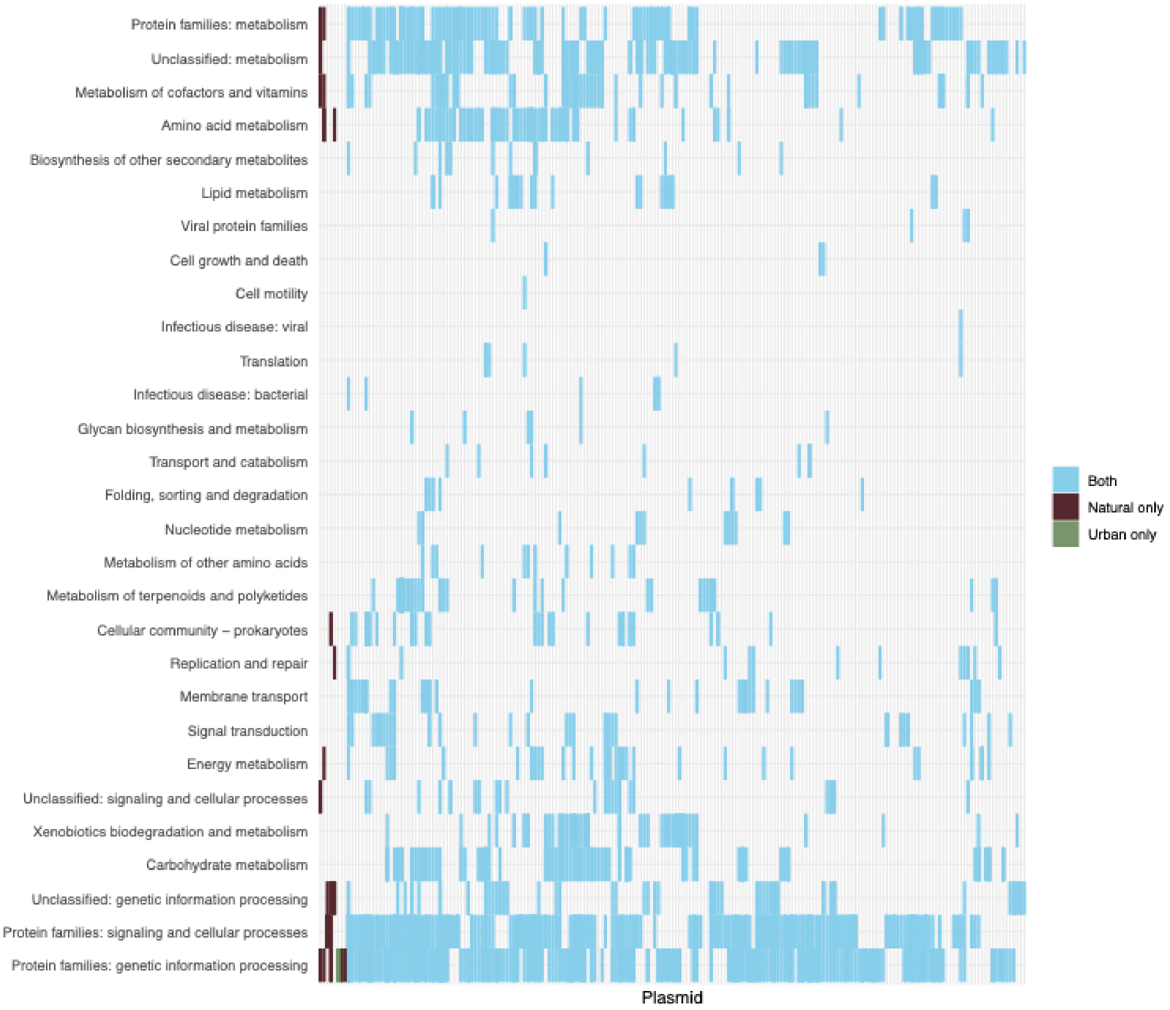

Supplementary Figure 4. Bacterial CRISPR investment. a. Bacterial CRISPR investment in urban greenspaces vs. natural arid soils (χ^2 = 0.33, p-value = 0.565). Individual data points are shown with jitter. The central point represents the mean, and error bars indicate ± one standard deviation. **b-c.** Relationships between the CRISPR

investment and relative abundance of plasmid taxonomic units (PTUs) and PTUs carrying antibiotic resistance genes.

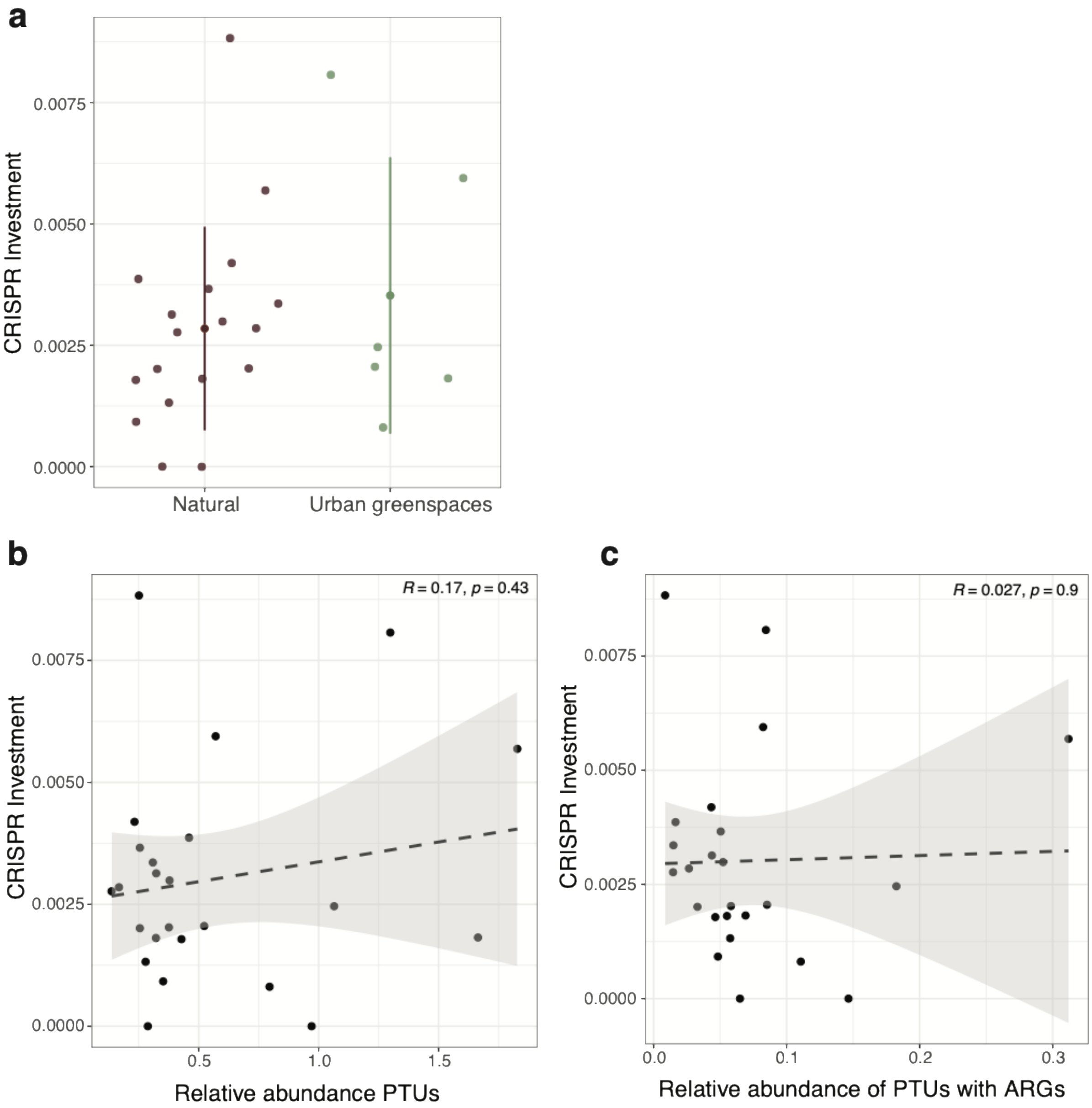

Supplementary Figure 5. Antibiotic resistance types. (a) Number of genes of resistance to each antibiotic class found in plasmid sequences**.**

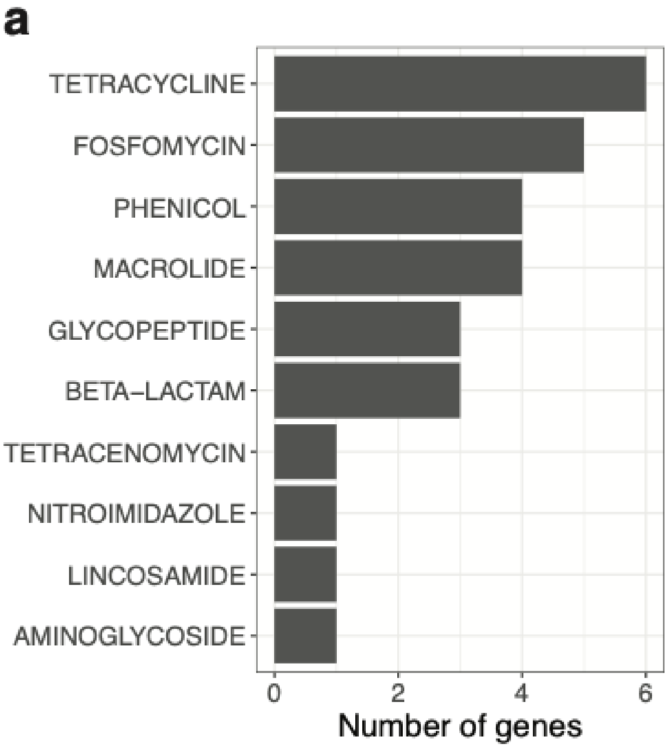

Supplementary Figure 6. Metal resistance types. (a) Number of genes of resistance to each heavy metal found in plasmid sequences.

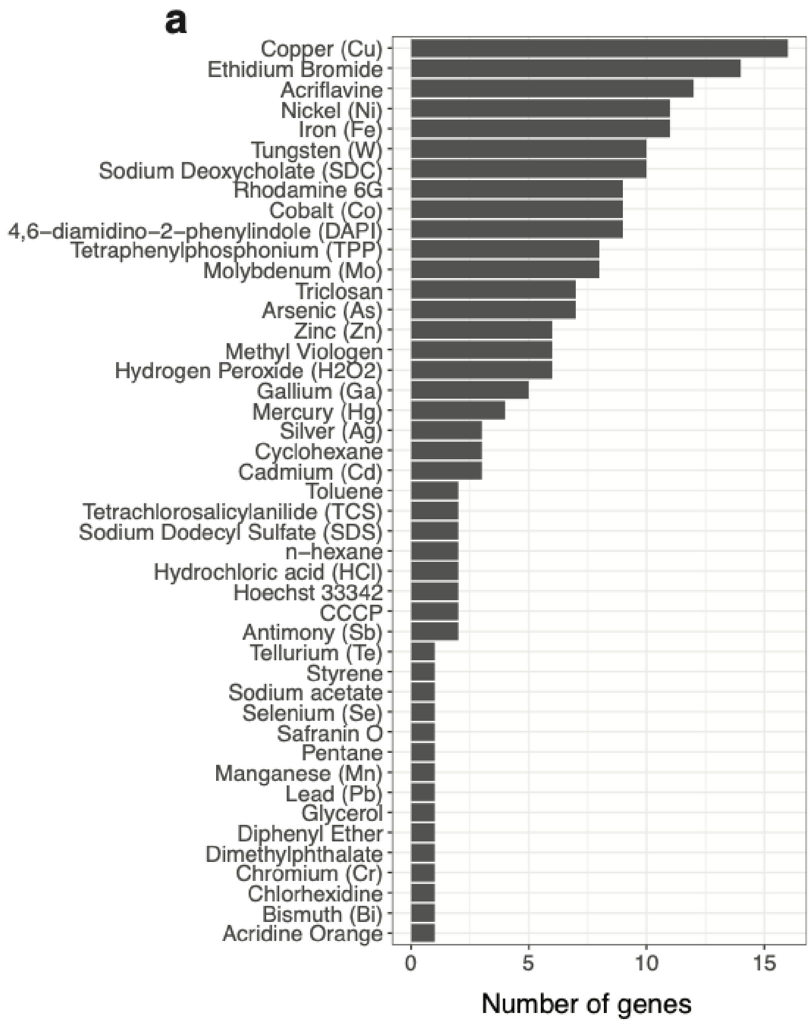

Supplementary Figure 7. Xenobiotic resistance types. (a) Number of genes of resistance to each xenobiotic found in plasmid sequences.

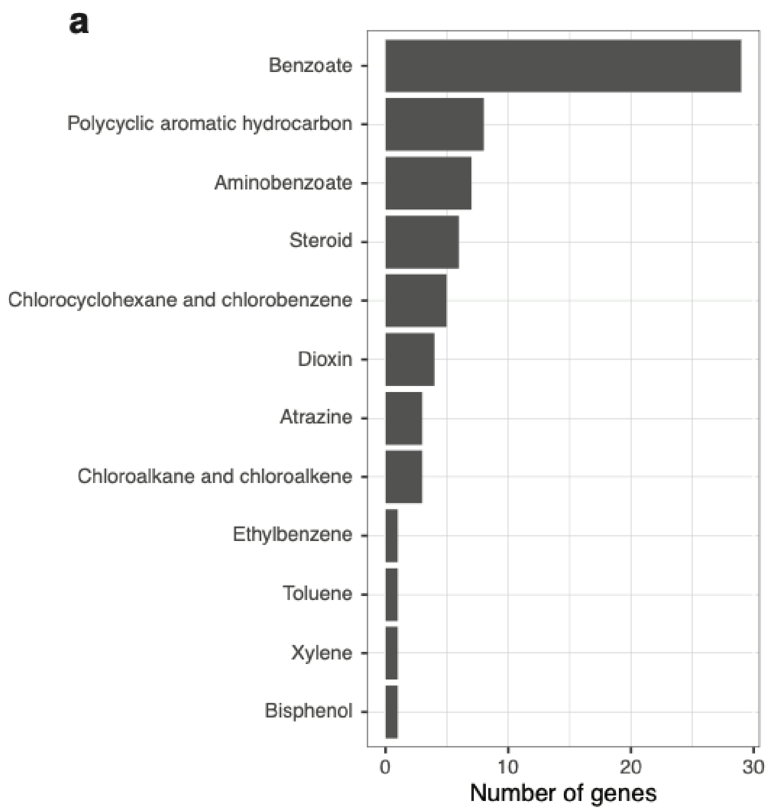

Supplementary Figure 8. PTU composition differences among natural soil types. A. NMDS plot (stress = 0.10) of Bray-Curtis dissimilarity matrices illustrating the differences in PTU composition between natural soil types (Sonoran Desert, arid shrubland and ponderosa pine forest). **B.** PCoA ordination of Bray-Curtis dissimilarity showing group

centroids (triangles) and individual sample dispersion (left), and boxplots of distances to centroids for each group (right).

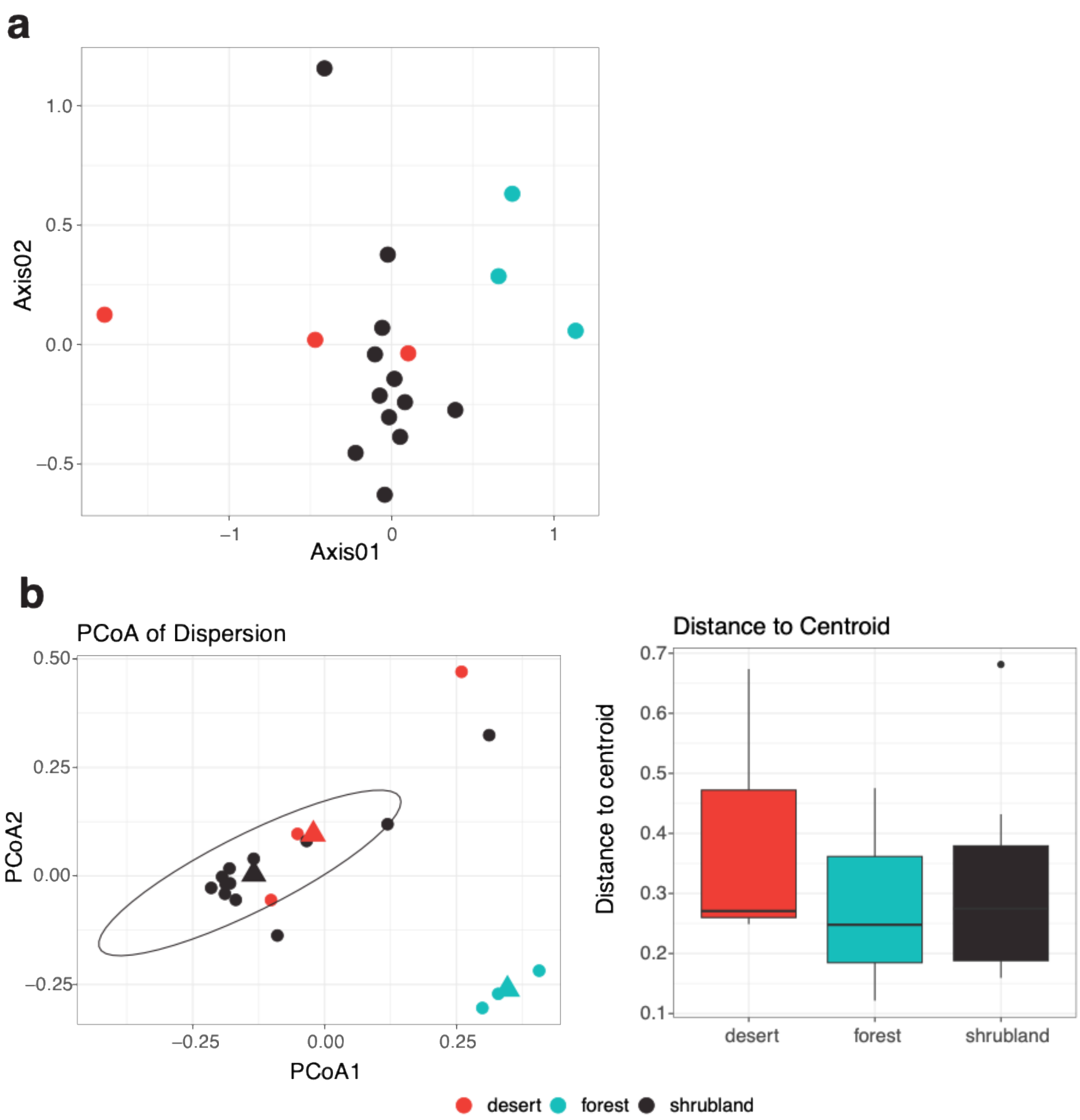

Supplementary Table 1. Sequence accession numbers.

Sample name	Urban.natural	Ecosystem	Site	Accession number
UAB-T1-9	Natural	Arid shrubland	UAB	SRX25578073
UAB-T1-6	Natural	Arid shrubland	UAB	SRX25578072
UAB-T1-3	Natural	Arid shrubland	UAB	SRX25578093
Ex45-T1-9	Natural	Arid shrubland	Ex45	SRX25578092
Ex45-T1-6	Natural	Arid shrubland	Ex45	SRX25578091
Ex45-T1-3	Natural	Arid shrubland	Ex45	SRX25578090
8-T1-9	Natural	Arid shrubland	8	SRX25578089
8-T1-6	Natural	Arid shrubland	8	SRX25578088
8-T1-3	Natural	Arid shrubland	8	SRX25578087
RP-T1-9	Urban	Urban greenspace	RP	SRX25578086

RP-T1-6	Urban	Urban greenspace	RP	SRX25578085
RP-T1-3	Urban	Urban greenspace	RP	SRX25578084
HP-T1-9	Urban	Urban greenspace	HP	SRX25578083
HP-T1-6	Urban	Urban greenspace	HP	SRX25578081
HP-T1-3	Urban	Urban greenspace	HP	SRX25578080
11B-T1-9	Natural	Arid shrubland	11B	SRX25578082
11B-T1-6	Natural	Arid shrubland	11B	SRX25578071
11B-T1-3	Natural	Arid shrubland	11B	SRX25578070
RC-T1-9	Natural	Ponderosa pine forest	RC	SRX25578079
RC-T1-6	Natural	Ponderosa pine forest	RC	SRX25578078
RC-T1-3	Natural	Ponderosa pine forest	RC	SRX25578077

SC-T1-9	Natural	Sonoran Desert	SC	SRX25578076
SC-T1-6	Natural	Sonoran Desert	SC	SRX25578075
SC-T1-3	Natural	Sonoran Desert	SC	SRX25578074
